# Chronic Striatal Cholinergic Interneuron Excitation Causes Cerebral Palsy-Related Dystonic Behavior in Mice

**DOI:** 10.1002/ana.27299

**Published:** 2025-07-03

**Authors:** Kat Gemperli, Xinguo Lu, Keerthana Chintalapati, Alyssa Rust, Rishabh Bajpai, Nathan Suh, Joanna Blackburn, Rose Gelineau-Morel, Michael C. Kruer, Dararat Mingbunjerdsuk, Jennifer O’Malley, Laura Tochen, Jeff L. Waugh, Steve Wu, Timothy Feyma, Joel Perlmutter, Steven Mennerick, Jordan G. McCall, Bhooma R. Aravamuthan

**Affiliations:** 1 Department of Neurology, Washington University School of Medicine, St. Louis, MO, USA; 2 Department of Psychiatry, Washington University School of Medicine, St. Louis, MO, USA; 3 Taylor Family Institute for Innovative Psychiatric Research, Washington University School of Medicine, St. Louis, MO, USA; 4 Department of Pediatrics, Northwestern University, Chicago, IL, USA; 5 Department of Pediatrics, University of Missouri-Kansas City School of Medicine, Kansas City, MO, USA; 6 Department of Child Health, University of Arizona College of Medicine Phoenix, Phoenix, AZ, USA; 7 Department of Neurology, Seattle Children’s Hospital, University of Washington, Seattle, WA, USA; 8 Department of Neurology and Neurological Sciences, Stanford University, Palo Alto, CA, USA; 9 Department of Neurology and Rehabilitation Medicine, George Washington School of Medicine and Health Sciences, Washington, DC, USA; 10 Department of Pediatrics, University of Texas Southwestern Medical Center, Dallas, TX, USA; 11 Department of Pediatrics, University of Cincinnati, Cincinnati, OH, USA; 12 Neurology, Gillette Children’s Specialty Healthcare, St. Paul, MN, USA; 13 Department of Anesthesiology, Washington University School of Medicine, St. Louis, MO, USA; 14 Center for Clinical Pharmacology, University of Health Sciences and Pharmacy in St. Louis and Washington University School of Medicine, St. Louis, MO, USA

## Abstract

**Objective:**

Mouse models of genetic dystonias have demonstrated abnormal striatal cholinergic interneuron excitability, but do not consistently demonstrate subjective dystonic features. To determine whether striatal cholinergic interneuron excitation can cause potentially dystonic motor behaviors, we first determined features correlated specifically with dystonia severity in people and then determined whether these features emerged in mice following striatal cholinergic interneuron excitation.

**Methods:**

Eight movement disorders experts rated dystonia severity in 193 videos of people with cerebral palsy doing a seated task. Leg adduction variability metrics, which are known to correlate with leg dystonia severity during gait, were quantified in these videos of seated tasks. Metrics significantly associated with leg dystonia severity during seated tasks in people were then quantified in mice and compared between mice who underwent chemogenetic striatal cholinergic interneuron excitation (n = 17) and mice who did not (n = 17).

**Results:**

Leg adduction variability correlated well with experts’ leg dystonia severity scores in people. Leg adduction variability was also significantly increased in mice that underwent striatal cholinergic interneuron excitation compared to mice that did not (*p* < 0.05). This difference was not present with acute excitation and emerged only after 14 days of ongoing excitation.

**Interpretation:**

We demonstrate that leg adduction variability correlates with leg dystonia severity in people with cerebral palsy and that chronic, but not acute, striatal cholinergic interneuron excitation can cause leg adduction variability in mice. These results support targeting striatal cholinergic interneurons for dystonia drug development and demonstrate the potential value of using quantifiable leg adduction metrics to study dystonia pathophysiology.

## Graphical Abstract

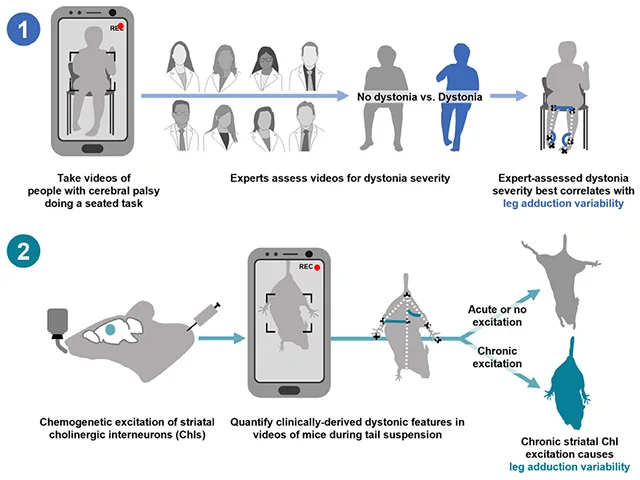

Dystonia is a functionally debilitating, painful and often medically refractory movement disorder characterized by variable overflow muscle activation triggered by voluntary movement.^1–5^ Mouse models of rare, monogenic, and progressive etiologies of dystonia have demonstrated abnormal excitability of striatal cholinergic interneurons (ChIs), suggesting a potential pathophysiologic link.^6–9^ However, it is unclear if excitation of striatal ChIs can independently cause a dystonic phenotype. Optogenetic excitation of striatal ChIs for several minutes does not produce dystonic behaviors in mice.^10^

Although early-onset dystonia pathophysiology has been most commonly studied in the context of rare monogenic progressive dystonia etiologies,^6,7,9–16^ the most common cause of early-onset dystonia is neonatal brain injury producing cerebral palsy (CP).^17–20^ In CP, dystonia appears after a temporal delay of months to years after the injury^3,21^ supporting the hypothesis that the underlying circuit pathology must persist for a threshold duration of time to yield dystonia.^22^ For example, long-term striatal ChI excitation beyond what has been assessed in optogenetic studies potentially could yield dystonic behavior in mice.

The definition of dystonic behavior in mice is, however, challenging. Clasping of the forelimbs or hindlimbs during tail suspension has been quantified in monogenic models of dystonia in mice,^6,7,23^ but extensive data suggests that clasping is not unique to dystonia in mice.^24,25^ Furthermore, clasping or other subjective dystonic behaviors have not been identified in many genetic mouse models of dystonia that recapitulate clinically relevant mutations.^26–28^ Clasping is also not a motor behavior directly related to dystonia in people.^29–31^

Electromyographic measures of antagonist muscle co-contraction have also been used to quantify dystonia in rodents.^14,32,33^ However, antagonist muscle co-contraction is not specific to dystonia alone and can be seen associated with other motor symptoms like spasticity,^34,35^ which often co-occurs with dystonia in CP and other conditions and requires a different treatment paradigm.^36,37^ Although not the only way to conceptualize dystonic behavior, one method to quantify behavior that may correspond to dystonia in mice is to identify quantifiable behaviors that correspond to dystonia in people.

Clinically, the gold standard for identification of dystonia in people relies on expert consensus-based assessment of physical exam videos.^30,31^ Our recent work has demonstrated that dystonia in the legs, the most common site of dystonia in people with CP, primarily manifests during gait as quantifiably variable leg adduction.^31,36^ It is unclear whether leg adduction variability tracks specifically with dystonia severity in people with CP during other tasks as well. If observed across tasks, and particularly if not correlated with leg spasticity severity, leg adduction variability may be a useful marker of motor dysfunction associated with leg dystonia in people with CP. Leg adduction variability may also serve as a quantifiable marker of motor dysfunction in mice that could be objectively tracked in response to striatal ChI excitation.

We hypothesized that leg adduction variability correlates specifically with leg dystonia severity in people with CP across tasks and that striatal ChI excitation would cause leg adduction variability in mice. To assess this, we first determined how leg adduction variability correlates with dystonia severity and spasticity severity in people with CP doing seated tasks. We next assessed whether chronic chemogenetic striatal ChI excitation in mice over 2 weeks causes hindlimb adduction variability during tail suspension.

## Materials and Methods

### Human Subjects and Video Collection

All human subject data collection was approved by the Washington University School of Medicine Institutional Review Board (IRB) with waiver of consent given that only clinically-acquired data was used for this study (IRB 202102101).

Subjects with CP were recruited from the Gillette Children’s Cerebral Palsy Center between January 1, 2020 and December 30, 2021. Of note, CP was chosen specifically because dystonia coexists with other movement symptoms in people with CP (most commonly spasticity). Inclusion criteria were that subjects had to be at least 3 years old and that they had to have a clinician-confirmed diagnosis of CP as per the 2006 consensus diagnostic criteria.^38^ Exclusion criteria were inadequate quality or content of the video recordings (eg, full body not visible for the task or the person was not able to complete the task during the video recording). Subjects were asked to do an alternating hand open/close task: they sat in a chair facing forward, rested the non-dominant hand on the ipsilateral thigh, and then raised and alternatingly opened and fisted the dominant hand as fast they were able for approximately 5 seconds. They, then, repeated this task with the non-dominant hand. Videos were recorded with a Panasonic AG-AC160AP camera at 1,920 × 1,080 pixel resolution (Panasonic, Osaka, Japan).

### Clinical Expert-Based Leg Dystonia Assessment in Human Subjects

For video assessment, faces of all subjects were blurred to ensure subject anonymity using ShotCut (Meltytech). Blurred videos were assessed for leg dystonia using the Global Dystonia Rating Scale (GDRS), a 10-point Likert scale score (0 = no dystonia, 10 = severe dystonia) that has demonstrated good inter-rater reliability for assessment of leg dystonia in people with CP.^31,39^ Three consensus-building data sets of 8 videos each were presented to 8 pediatric movement disorders physicians with expertise in CP practicing at different institutions across the United States (J.B., R.G.M., M.K., D.M., J.O.M., L.T., J.W., S.W.) This group of reviewers met for three 1-hour Zoom-based consensus-building discussions to ensure shared understanding and application of the GDRS for leg dystonia rating in CP (Zoom Video Communications).

Of note, all reviewers were told that subjects had CP and that most had spasticity. We asked reviewers to explicitly grade the severity of uniquely dystonic movements and not spasticity or other motor symptoms. To establish initial consensus among the group regarding the features that would be graded as dystonia, 24 videos were assessed by all 8 reviewers across these 3 consensus-building sessions. During these sessions, experts described features of variable leg adduction as guiding their leg dystonia assessments, recapitulating previous work demonstrating that expert reports of variable leg adduction guide leg dystonia identification and severity assessment in young people with CP.^30,31^ The remaining videos were independently assessed for leg dystonia and scored using the GDRS by 2 of the 8 experts. Each expert was assigned videos across a range of possible dystonia severities. For any videos where the total leg dystonia GDRS score differed by more than 6 between the 2 reviewers, the whole group of 8 experts was asked to assess the video. The average score across the 2 reviewers, or across all 8 reviewers for the consensus building video data set or for videos with scoring discrepancies, were used for final analysis.

### Mouse Tail Suspension Task and Video Collection

All experiments and procedures were approved by the Institutional Animal Care and Use Committee of Washington University School of Medicine in accordance with National Institutes of Health guidelines.

Mice were from litters resultant from breeding choline acetyl-transferase-Cre (ChAT-Cre, Jackson Laboratory Strain 006410, Jackson Labs, Bar Harbor, ME) mice with wild-type C57/Bl6J (Jackson Laboratory Strain 000664) mice. Both sexes were used for all experiments. Mice were held 1 inch above the tail base and suspended 1 foot above a table surface in front of a solid colored background for 1 min. Videos of the ventral side of the mouse were recorded using a Google Pixel 2 (Google, Mountain View, CA) on a tripod placed 6 inches in front of the mouse. Videos were recorded at 120 frames per second at 1,980 × 1,020 pixel resolution.

### Video-Based Pose Estimation and Calculation of Dystonia Metrics

DeepLabCut^40,41^ (https://github.com/DeepLabCut/) was used to determine lower limb positioning in videos. DeepLabCut allows for the user to label a subset of video frames for points of interest and then trains a neural network based on that manual labeling to subsequently label the remaining frames for those points of interest. DeepLabCut does not allow for the determination of the presence or absence of a behavior itself, only the body part locations in each video frame.

Noting that variable leg adduction has been described as a key feature of leg dystonia during gait in people with CP and has been shown to correlate linearly with expert-assessed leg dystonia severity,^30,31^ leg and hindlimb features that allow for quantification of leg adduction variability were determined in each video frame.

In human subjects, the midpoint of the patella (knee), midpoint between the medial and lateral malleolus (ankle), and tip of the third toe were labeled on both legs. These points were manually labeled on 10 frames extracted from each video and used to train a ResNet-50-based neural network for 1,030,000 iterations with 95% of frames used for training and 5% of frames used for testing the network to yield less than a 5 pixel train error and 5 pixel test error.

In mice, the hindpaw heels, the tip of the hindpaw third toes, anus, the trunk midpoint between the 2 hindlimbs, and the nose tip were labeled. These points were manually labeled on 10 frames extracted from 30 videos taken on different experimental days and from different experimental cohorts. These manually labeled frames were used to train another network as described above.

Points labeled using these trained networks were conditioned at a p-cutoff of 0.99 to ensure only accurately labeled points were used for further analysis.

The variances (corresponding to adduction variability) and minima (corresponding to adduction amplitude) were calculated for the following leg/hindlimb adduction metrics using custom written code in MATLAB (R2021, The Mathworks, Natick, MA). (1) For human subjects, the foot angle (between the knee, ankle, and toe), inter-knee distance, inter-ankle distance, and inter-toe distance, with distance metrics calculated along an X-axis parallel to the floor and divided by the maximum knee-to-ankle distance to allow comparison across videos. (2) For mice, the foot angle (between the anus, heel, and toe), leg angle (between the trunk midpoint, anus, and heel), inter-heel distance, and inter-toe distance, with distance metrics calculated perpendicular to midline and divided by the maximum nose-to-anus distance to allow comparison across videos.

The MATLAB code, representative videos, and trained DeepLabCut labeling models are available here: https://wustl.box.com/s/07agml8jmw681et1bnsk2j4atghy5j9l.

### Chemogenetic Excitation of Striatal ChIs in Mice

Striatal ChIs are abnormally excitable in genetic mouse models of dystonia,^8,9^ are increased in a rat model of cerebral palsy and dystonia,^42^ and are preferentially lost in the dorsal striatum in a genetic mouse model of DYT1.^7,43^ To determine whether chronic striatal ChI excitation could cause leg adduction behavior in mice, we targeted expression of excitatory Gq designer receptors exclusively activated by designer drugs (DREADDs) to the dorsal striatum using stereotaxic injections of AAV8-hSyn-DIO-hM3d(Gq)-mCherry (Addgene, Watertown, MA, catalog: 44361-AAV8). To target these receptors to ChIs, we used ChAT-Cre mice on a C57/Bl6J background (Jackson Labs Strain 006410) that were heterozygously bread with wild-type C56/Bl6J mice (Jackson Labs Strain Strain 000664). The experimental group (ChAT-Cre positive mice) expressed these excitatory DREADDs in ChIs in the dorsal striatum, while the control group (ChAT-Cre negative littermates) did not. Mice were genotyped via tail snip at post-natal day 28.

Between 5 and 6 months of age, all mice were stereotaxically injected under isoflurane anesthesia (1%v/v in oxygen, 1L/min) with 250nL of AAV8-hSyn-DIO-hM3d(Gq)-mCherry at a rate of 50nL/min using a 1,000nL Hamilton syringe bilaterally in the dorsal striatum (coordinates: +1.0mm anterior and ± 1.5mm lateral to bregma, 2.7mm deep to the cortical surface). Following 21 days of recovery and allowance for excitatory Gq receptor expression, mice underwent a 14-day tail suspension and administration protocol of the selective Gq receptor agonist clozapine-N-oxide (CNO) (watersoluble, HelloBio, Princeton, NJ, catalog: HB6149). On day 0, mice were habituated to handling and the tail suspension procedure. Mice were assessed for leg adduction behavior during tail suspension on days 1, 7, and 14 as described above. On day 1, mice were injected intraperitoneal (i.p.) with 1mg/kg of CNO 30 minutes before tail suspension. Mice were then given CNO continuously via their drinking water (1mg/kg/day) through day 14 behavioral analysis. On days 7 and 14, mice were again given 1mg/kg i.p. injection of CNO 30 minutes before tail suspension, to ensure comparability in exposures between each day of the behavioral paradigm (ie, injections are received by the mice before tail suspension on each day) and to ensure that a full day’s dose of CNO was delivered to these animals on the day of behavior regardless of any variation in water consumption.

### Drug Administration

The anticholinergic agent trihexyphenidyl (THP) is clinically variably effective for the treatment of dystonia, particularly in those with CP,^5,44^ but it remains commonly used^45^ and has been shown to improve motor performance in mouse models of the genetic dystonias like DYT1.^14,46,47^ Therefore, in addition to determining whether chemogenetic striatal ChI excitation would cause leg adduction behavior in mice, we additionally determined whether THP would reduce this behavior.

As described above, ChAT-Cre mice were stereotaxically injected with 250nL of AAV8-hSyn-DIO-hM3d(Gq)-mCherry bilaterally in the dorsal striatum. Noting that THP must be administered chronically over several weeks to have optimal clinical effect and given our hypothesis that striatal ChI excitation may need to occur chronically to yield a dystonic phenotype, we began administering THP 0.8mg/kg^48–50^ (Fisher Scientific, Waltham, MA catalog: T33035G) or vehicle intraperitoneally daily beginning the day following intracranial viral injection. The vehicle required to fully dissolve THP in solution was 1% ethanol, amounting to 80mg/kg/day, a dose that has been shown to not have locomotor effects in mice.^48^ As described above, 21 days after surgery, mice underwent a 14-day tail suspension protocol and begin additionally receiving CNO to selectively excite the striatal ChIs. Behavior during tail suspension was compared between mice undergoing striatal ChI excitation who received THP or the 1% ethanol vehicle.

### Mouse Brain Slice Preparation and Cell-Attached Recordings

To identify ChIs in brain slices, striatal ChI firing rates were compared between groups of ChAT-Cre positive mice injected with either AAV8-hSyn-DIO-hM3d(Gq)-mCherry (ChIs would express mCherry and the Gq receptor and be excited by CNO) or injected with AAV8-hSyn-DIO-mCherry (Addgene catalog: 50459-AAV8, ChIs would express only mCherry and not be excited by CNO) as described above. All mice underwent the same CNO exposure paradigm as mice undergoing behavioral assessment. Striatal ChI firing rates were assessed in slice preparations on day 1 and day 14 of this CNO exposure paradigm, at least 6 hours after the last exposure to CNO.

For slice preparation, mice were anesthetized with isoflurane and decapitated according to protocols approved by the Washington University Institutional Animal Care and Use Committee. The brain was removed from the skull and attached to a Leica VT1200 specimen holder. Coronal brain slices (300μm thick) were cut in ice-cold, modified NMDG-HEPES recovery aCSF (in mM: 92 NMDG, 2.5 KCl, 1.25 NaH_2_PO4, 30 NaHCO3, 20 HEPES, 25 glucose, 2 thiourea, 5 Na-ascorbate, 3 Na-pyruvate, 0.5 CaCl_2_, and 10 MgSO_4_; 300 mOsm; pH 7.3–7.4). Slices were transferred into modified NMDG-HEPES recovery aCSF at 32°C. A Na^+^-rich spike-in solution (4 ml, 2 M) was added to gradually increase Na^+^ concentration to improve the success of gigaseal formation. After recovery, slices were kept in modified HEPES holding aCSF (in mM: 92 NaCl, 2.5 KCl, 1.25 NaH_2_PO_4_, 30 NaHCO_3_, 20 HEPES, 25 glucose, 2 thiourea, 5 Na-ascorbate, 3 Na-pyruvate, 2 CaCl_2_, and 2 MgSO_4_; 300 mOsm; pH 7.3–7.4) for at least 1 hour at room temperature before experimental recording.

For recording, slices were transferred to a recording chamber with continuous perfusion (2ml/min, 32°C) of oxygenated, Na^+^-rich aCSF (in mM: 125 NaCl, 25 glucose, 25 NaHCO_3_, 2.5 KCl, 1.25 NaH_2_PO_4_, equilibrated with 95% oxygen-5% CO_2_ plus 2 CaCl_2_, and 1 MgCl_2_, 310 mOsM). Target interneurons were labeled and identified by fluorescence. IR-DIC microscopy (Nikon FN1 microscope and Photometrics Prime camera) was used to visualize fluorescent cells for pipette attachment. A MultiClamp 700B amplifier, Digidata 1,550 16-bit A/D converter, and pClamp 10.4 software (Molecular Devices) were used for recordings. To form a loose-seal, cell-attached patch, Borosilicate glass pipettes (World Precision Instruments), filled with regular aCSF, had an open-tip resistance of 3 to 6MΩ. Action potential activity was recorded in current-clamp mode with sampling rate at 20kHz and filtered at 6kHz.

### Mouse Brain Immunohistochemistry and Quantification

Mice were deeply anesthetized with isoflurane and transcardially perfused with 1× phosphate-buffered saline (PBS) followed by 4% paraformaldehyde solution. Extracted brains were placed in 4% paraformaldehyde solution overnight and were stored in 30% sucrose until further processing. Before slicing and staining, brains were treated overnight with 20% glycerol and 2% dimethylsulfoxide to prevent freeze-artifacts. The brains were rapidly frozen, after curing by immersion in 2-methylbutane chilled with crushed dry ice and mounted on a freezing stage of an AO 860 sliding microtome and sliced into 20 μM coronal sections collected in Antigen Preserve solution (50% PBS pH 7.0, 50% ethylene glycol, 1% polyvinylpyrrolidone). For immunofluorescent histochemistry, free floating sections were stained for ChAT (primary: ChAT fluoro stain, Millipore ab144p; secondary: anti-goat AlexaFluor 647, Jackson Labs 705-605-147) and mCherry (primary: mCherry Double Stain - ChAT/f, EnCor RPCA-mCherry; secondary: anti-rabbit Cy3, Jackson Labs 711-165-147). All incubation solutions from the primary antibody onward use Tris buffered saline (TBS) with Triton X-100 as the vehicle; all rinses are with TBS.

### Striatal DREADD Expression Quantification

To determine the extent of DREADD expression in striatal ChIs, mounted sections were imaged using a Keyence BZ-X810 fluorescence microscope (Keyence Corporation of America, Itasca, IL). For each slice ranging from 145 to 945μm anterior to bregma, each panel containing striatum was imaged with a 10^ objective using the multi-color setting to capture an overlay of ChAT and mCherry co-expression and image stitch in the BZ-X800 Analyzer to generate a full, uncompressed image of each striatal section. To count the total number of ChIs in each striatal section, we converted all images to grayscale (8-bit) in ImageJ with a minimum default threshold of 10. We processed the resulting selection using watershed segmentation and then used the built-in analyze particles function, setting the minimum particle size to 9 pixels, to count the total number of selected neurons within the striatum region within the slice. To determine the number DREADD-expressing ChIs in each striatal section, the neurons expressing both ChAT and mCherry were manually counted in each slice and cross-referenced to neurons identified with automated counting to ensure any neurons that were manually identified were also included in the total calculated ChI population. For any given slice, the percentage striatal ChIs expressing the DREADD was calculated by dividing the number of mCherry-expressing ChIs by the total number of ChIs.

### Statistical Analyses

Statistical analysis was done in GraphPad Prism (version 8, GraphPad Software), SPSS (version 28.0, IBM), or within an Anaconda virtual environment with the following packages for feature selection analyses; python 3.9.16, scikit-learn 1.2.2, scipy 1.10.0, and mrmr-selection 0.2.6. Leg adduction variability and amplitude metrics in human subjects were compared between videos with and without expert-identified dystonia using *t* tests when appropriate (with normality of data sets assessed using the Shapiro–Wilk test). For data with unequal standard deviations between groups, a t test with Welch’s correction was applied. For data that were not normally distributed, Mann–Whitney tests were used. Pearson correlations and multiple linear regression were used to determine whether these leg adduction metrics were significantly correlated to expert assessments of dystonia severity. For determining which of the 8 variability and amplitude metrics best discriminate between the presence or absence of dystonia or predicted dystonia severity in human subjects, 7 filter, wrapper, and embedded feature selection methods were used (Spearman’s ρ, Kendall’s τ, univariate feature selection, maximum relevance–minimum redundancy, extra trees classifier, random forest, and sequential feature selection [forward]).^49^ Two-way repeated measures analysis of variance (ANOVAs) were used to determine whether there were significant differences in leg adduction variability and amplitude metrics in mice in response to acute versus chronic CNO administration and in response to THP versus vehicle administration. *t* Tests and paired *t* tests were used to assess differences in striatal ChI firing rate between mice either expressing or not expressing DREADDs (*t* test) or in cells before and after CNO (paired *t* test). The significance level for all tests was set a priori to *p* < 0.05.

## Results

### Leg Dystonia Severity Is Primarily Associated with Leg Adduction Variability in People with CP

Experts noted that 104 of 193 people assessed (demographic characteristics in Table) had some degree of leg dystonia, comparable to published rates of dystonia in people with CP.^30,31,36^ Fifty-two of 104 had mild dystonia (GDRS, 1 to <4), 25 of 104 had moderate dystonia (GDRS, 4 to <7), and 27 of 104 had severe dystonia (GDRS, 7 or more). Almost all calculated metrics of leg adduction amplitude and variability were significantly greater in those with expert-identified leg dystonia compared to those without (*t* test, *p* < 0.05) (Fig 1). Almost all of these metrics also correlated with GDRS leg dystonia severity score (Pearson correlation, *p* < 0.01) (Fig 2).

To determine which quantified metrics served as the best objective measures of dystonia, we used multiple linear regression models, receiver-operator-characteristic curves, and multiple feature selection methods. These all showed that leg adduction variability better related to dystonia severity than did leg adduction amplitude. A multiple linear regression model relating dystonia severity to only the 4 variability metrics had an adjusted R^2^ of 0.546 (F, 57.6; *p* < 0.001), whereas a model including just the 4 amplitude metrics had a lower adjusted R^2^ of 0.268 (F, 18.2; *p* < 0.001). Including all 8 assessed metrics of variability and amplitude in a multiple linear regression only marginally improved the adjusted R^2^ to 0.584 (F, 34.0; *p* < 0.001) compared to only including the variability metrics. Furthermore, metrics related to variability were best able to discriminate between dystonia and no dystonia as indicated by receiver-operator-characteristic curves, with 3 of the 4 variability metrics demonstrating the highest areas under the curves (Fig 3). Finally, we used 7 filter, wrapper, and embedded feature selection methods to either assess how well variability or amplitude metrics can discriminate between the presence or absence of dystonia or predict dystonia severity. All 7 of these methods selected variability metrics more often than amplitude metrics as the primary contributors to models either discriminating between the presence or absence of dystonia or models predicting dystonia severity (Table S1). Taken together, these data suggest that leg amplitude variability in people with CP relates to dystonia severity better than amplitude metrics.

### Leg Adduction Variability Increases with Chronic Striatal ChI Excitation in Mice, but Not when Additionally Treated with Ethanol or Trihexyphenidyl Administration

We next sought to determine how these leg adduction variability metrics, which correlate with expert-assessed leg dystonia severity, may be leveraged to understand the effects of striatal ChI excitation and potential dystonia pathophysiology in mice. First, we established that DREADD expression co-localized to ChAT expression in the dorsal striatum in the experimental group (Fig 4A). We also saw that a relatively small percentage of the total striatal ChIs in any given slice were labeled (2–18% of striatal ChI cell bodies visible on any slice between 145 and 945μm anterior to bregma) (Fig 4B).

To validate that striatal ChIs were being excited with the DREADD agonist CNO, we made cell attached recordings from striatal ChIs expressing either the excitatory Gq DREADD or a fluorescent mCherry reporter (Fig 5A). As demonstrated previously, striatal ChIs expressing the Gq DREADD had a significantly higher baseline firing rate (*t* test, *p* < 0.05), which was potentiated with CNO (paired *t* test, *p* < 0.05) (Fig 5B, C).^50^ This was observed in mice on day 1 and day 14 of ongoing CNO exposure (Fig 5C), demonstrating that mice in the experimental group were undergoing chronic striatal ChI excitation during the behavioral paradigm, while mice in the control group were not.

Despite targeting only a small proportion of striatal ChIs, sustained excitation of these neurons over 14 days with repeated administration of CNO (Fig 6A) increased leg adduction variability during tail suspension. Foot angle variance, leg angle variance, and variance of the distance between the hindpaw heel and trunk midline increased over time and significantly differed between the control (n = 17) and experimental groups (n = 17) by day 14 of ongoing striatal ChI excitation (Fig 6C) (2-way repeated measures ANOVA, *p* < 0.05). However, there was no significant difference between groups on day 1 of behavioral assessment, suggesting that chronic striatal ChI excitation over a period of several days is required to yield this leg adduction behavior. There was no significant effect on leg adduction amplitude during tail suspension. Daily treatment with 1% ethanol (80mg/kg/day) with or without THP at 0.8mg/kg/day prevented increases in leg adduction variability with ongoing striatal ChI excitation (Fig 6).

Importantly, the changes in leg adduction variability were not solely because of increased leg movement. Total movement of the hindpaw (measured at the heel and toe relative to the trunk midpoint) did not change in mice undergoing striatal ChI excitation or undergoing ethanol or THP treatment across the whole 14 day span (Fig S1).

## Discussion

We have demonstrated that expert assessment of leg dystonia severity, not spasticity severity, correlates with leg adduction variability and that leg adduction variability emerges after chronic, but not acute, striatal ChI excitation in mice. We have additionally demonstrated that administration of ethanol with or without an anticholinergic agent abolishes leg adduction variability following chronic striatal ChI excitation.

Abnormal striatal ChI excitability has been found in many mouse models of genetic dystonias.^8,9^ The loss of these neurons in the dorsal striatum temporally relates to the onset of dystonic symptoms in a mouse model of DYT1.^7^ Yet, a causative link remained elusive, noting that previous work has examined behavioral outcomes after exciting striatal ChIs in mice for only minutes.^10^

We demonstrate that striatal ChI excitation has to occur over a prolonged period of time (at least 14 days of continuous excitation) to yield leg adduction behavior in mice. This finding has potential clinical implications. It has long been established that dystonia emerges following a delay of months to years after a brain injury.^3,21^ Furthermore, people with dystonia respond best to surgical treatments (ie, deep brain stimulation) if their dystonia is treated early.^51,52^ These results potentially suggest that reducing striatal ChI excitation in the period following brain injury, but before the emergence of dystonia, may mitigate or possibly prevent dystonia. At the very least, these results support high vigilance for early diagnosis of dystonia so that early treatment (pharmacologic or otherwise) can be considered.

Concomitant administration of 80mg/kg/day of ethanol with or without 0.8mg/kg/day of trihexyphenidyl prevents increased leg adduction variability following striatal ChI excitation. Of note, this dosing of ethanol is below established thresholds for causing general locomotor effects,^48^ as we have also confirmed (Fig S2). Although trihexyphenidyl is often used to manage dystonia in CP, the most commonly used medications to manage dystonia in CP are GABAergic agents like baclofen and benzodiazepines.^45^ The GABAergic effects of ethanol may be what prevents leg adduction behavior from emerging in mice following striatal ChI excitation, although this requires further study with more targeted GABAergic agents compared against a vehicle that does not have GABAergic effects itself. Future work should assess whether delayed treatment (as opposed to preventative concomitant treatment) with GABAergic or anticholinergic agents can reduce leg adduction variability in mice after striatal ChI excitation.

It is notable that striatal ChI excitation in mice expressing Gq DREADDs appeared to occur at baseline even before CNO administration, as demonstrated previously.^50^ This suggests that the time window of ongoing striatal ChI excitation required to yield dystonia is at least 14 days, but may be longer (given that we waited 21days to allow DREADD expression before beginning to assess behavior). Future work could assess whether exogenous expression of Gq DREADDs in striatal ChIs in isolation, without administration of a DREADD agonist, would result in dystonic behavior in mice. However, even noting that striatal ChIs increase their firing rate with expression of the Gq DREADD alone, these data support that chronic striatal ChI excitation is necessary to cause dystonic behavior in mice, in line with the clinically observed delay between an inciting brain injury and resultant dystonic behavior in people.

Our results may also inform assessment of leg dystonia in people with CP. Dystonia most commonly presents in the legs in people with CP^37^ and typically begins in the legs in people with the most common monogenic form of dystonia (DYT1).^5,53–55^ The legs are also where dystonia is most commonly under diagnosed.^37^ We have previously established that leg adduction variability correlates with leg dystonia severity during gait in people with CP.^30,31^ However, using gait alone as the task to facilitate dystonia identification requires assessment outside of a standard clinical exam room and also precludes the assessment of non-ambulatory individuals. Here, we show that leg adduction variability can still be used to assess individuals specifically for leg dystonia during seated upper extremity tasks, and that this leg adduction variability does not correlate with measures of spasticity. Therefore, leg adduction variability may be valuable specifically for leg dystonia assessment in people with CP as it has been demonstrated across 2 large cohorts of individuals across 2 different motor tasks.^30,31^

This study established that leg adduction variability tracks with expert consensus-based severity assessments of leg dystonia, not spasticity, but only in people with CP. Although CP is the most common cause of early-onset dystonia, and although CP has multiple etiologies,^17,19,20^ CP may not necessarily represent the way dystonia manifests across all possible etiologies. A similar approach should be tested in people with rarer etiologies of dystonia.

Because leg adduction variability correlates with leg dystonia severity in people with CP, we suggest that leg adduction variability could potentially be a marker of dystonic behavior in mice. We assessed mice for leg adduction metrics during the task typically used to assess dystonia (tail suspension), but assessing mice for dystonia metrics across other tasks may support the translational relevance of leg adduction variability as a dystonia analog. To this end, we have recently demonstrated that mice born preterm (a common CP etiology) demonstrate leg adduction variability during gait.^56^ However, it is important to recognize that behavioral correlates of dystonia in people may not necessarily represent behavioral correlates of dystonia across species. Quantifying the behaviors that specifically distinguish mouse models of monogenic or “pure” dystonias from mouse models of other motor dysfunction, perhaps by using the pose estimation techniques used in this study, could help further establish signatures of dystonic behavior specific to mice.

## Supplementary Material

Table S1

Additional supporting information can be found in the online version of this article.

## Figures and Tables

**FIGURE 1:**
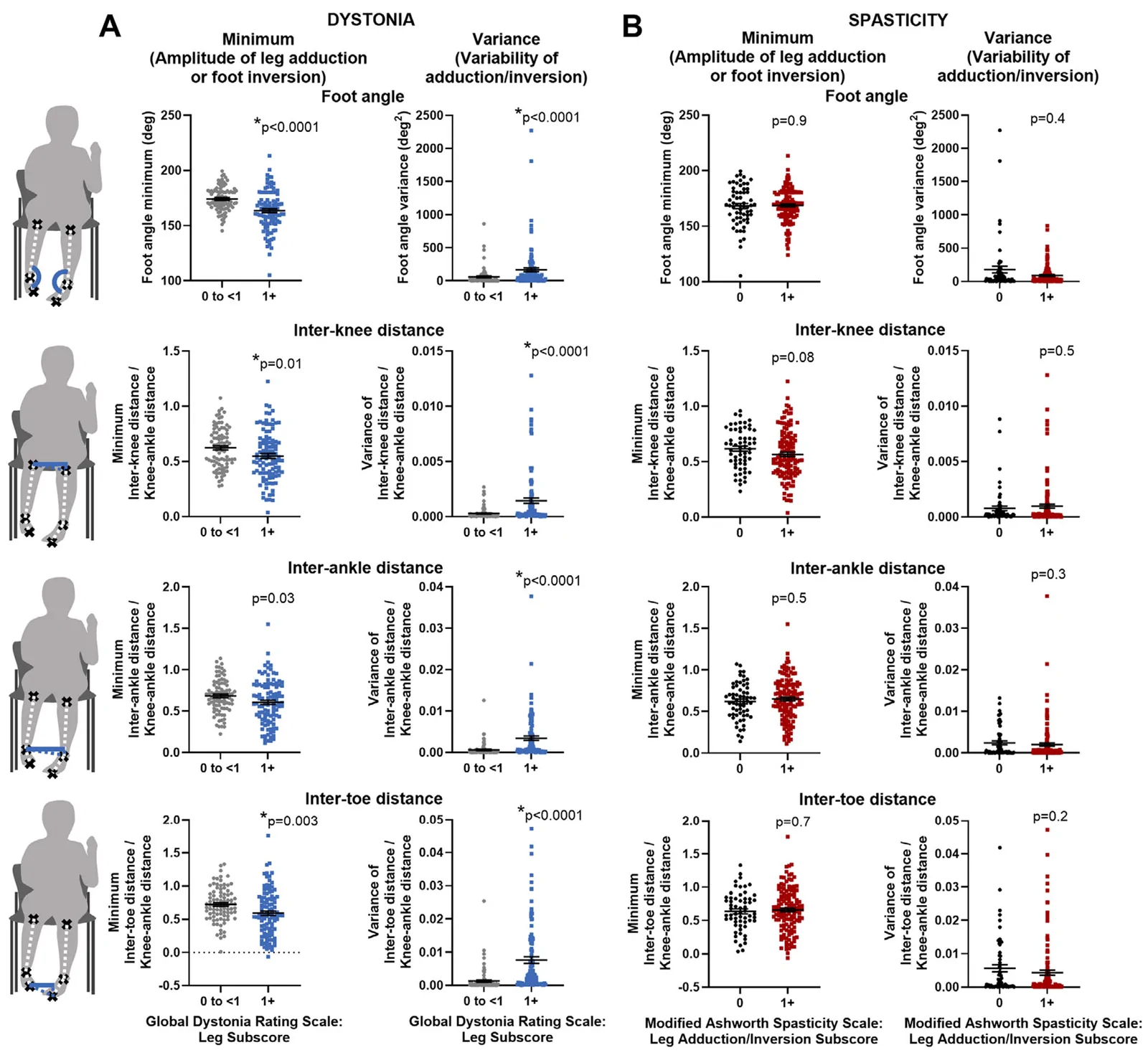
Differences in leg adduction variability and amplitude between people with and without leg dystonia or leg spasticity. (A) Metrics of leg adduction amplitude (left column) and variability (right column) are compared between people with cerebral palsy (CP) and leg dystonia (blue, n = 103, average Global Dystonia Rating Scale [GDRS] of 1 or more) and without leg dystonia (gray, n = 90, average GDRS of 0 to <1). Metrics are the minima (left column) and variances (right column) of the foot angle in the coronal plane (top row: angle between the knee, ankle, and toe labels), inter-knee distance (second row), inter-ankle distance (third row), and inter-toe distance (bottom row) with distance metrics calculated along an X-axis parallel to the floor and divided by the maximum knee-to-ankle distance to allow comparison across videos. (B) The same metrics of leg adduction amplitude and variability are compared between people with CP and leg adduction/inversion spasticity (red, n = 131, Modified Ashworth Score for hip adduction and foot inversion of 1 or more) and without leg adduction/inversion spasticity (black, *n* = 62, Modified Ashworth Score for hip adduction and foot inversion of 0). Comparisons are by *t* test, *p < 0.05. [Color figure can be viewed at www.annalsofneurology.org]

**FIGURE 2:**
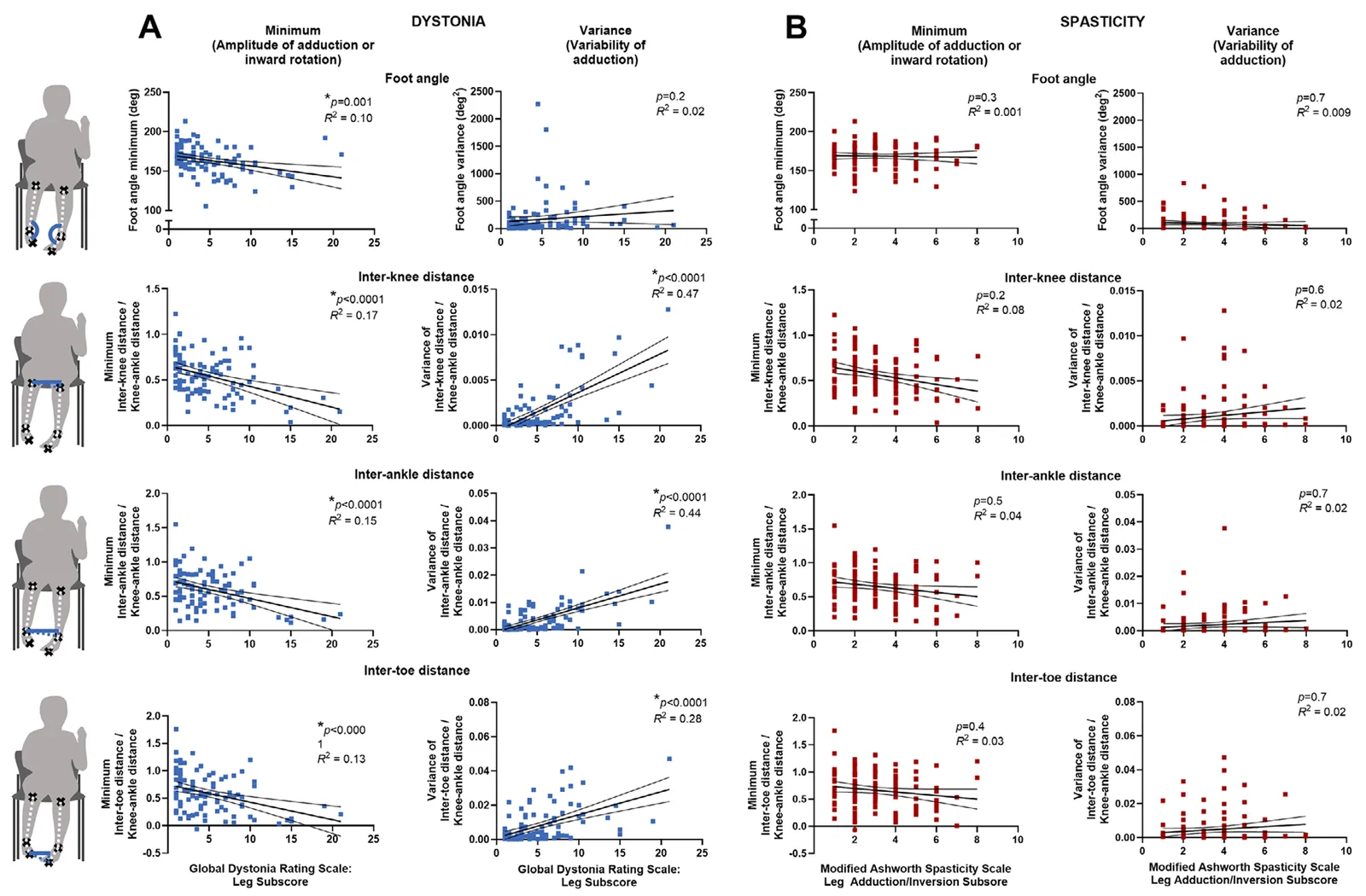
Correlation between leg dystonia and spasticity severity and leg adduction variability and amplitude. (A) Metrics of leg adduction amplitude (left column) and variability (right column) are correlated with average Global Dystonia Rating Scale (GDRS) scores indicated leg dystonia severity in people with cerebral palsy (CP) and leg dystonia (blue, n = 103, average GDRS of 1 or more). Metrics are the minima (left column) and variances (right column) of the foot angle in the coronal plane (top row: angle between the knee, ankle, and toe labels), inter-knee distance (second row), inter-ankle distance (third row), and inter-toe distance (bottom row) with distance metrics calculated along an X-axis parallel to the floor and divided by the maximum knee- to-ankle distance to allow comparison across videos. (B) The same metrics of leg adduction amplitude and variability correlated with leg adduction/inversion spasticity severity (red, n = 131, Modified Ashworth Score for hip adduction and foot inversion of 1 or more). Pearson correlation, *p < 0.05. [Color figure can be viewed at www.annalsofneurology.org]

**FIGURE 3:**
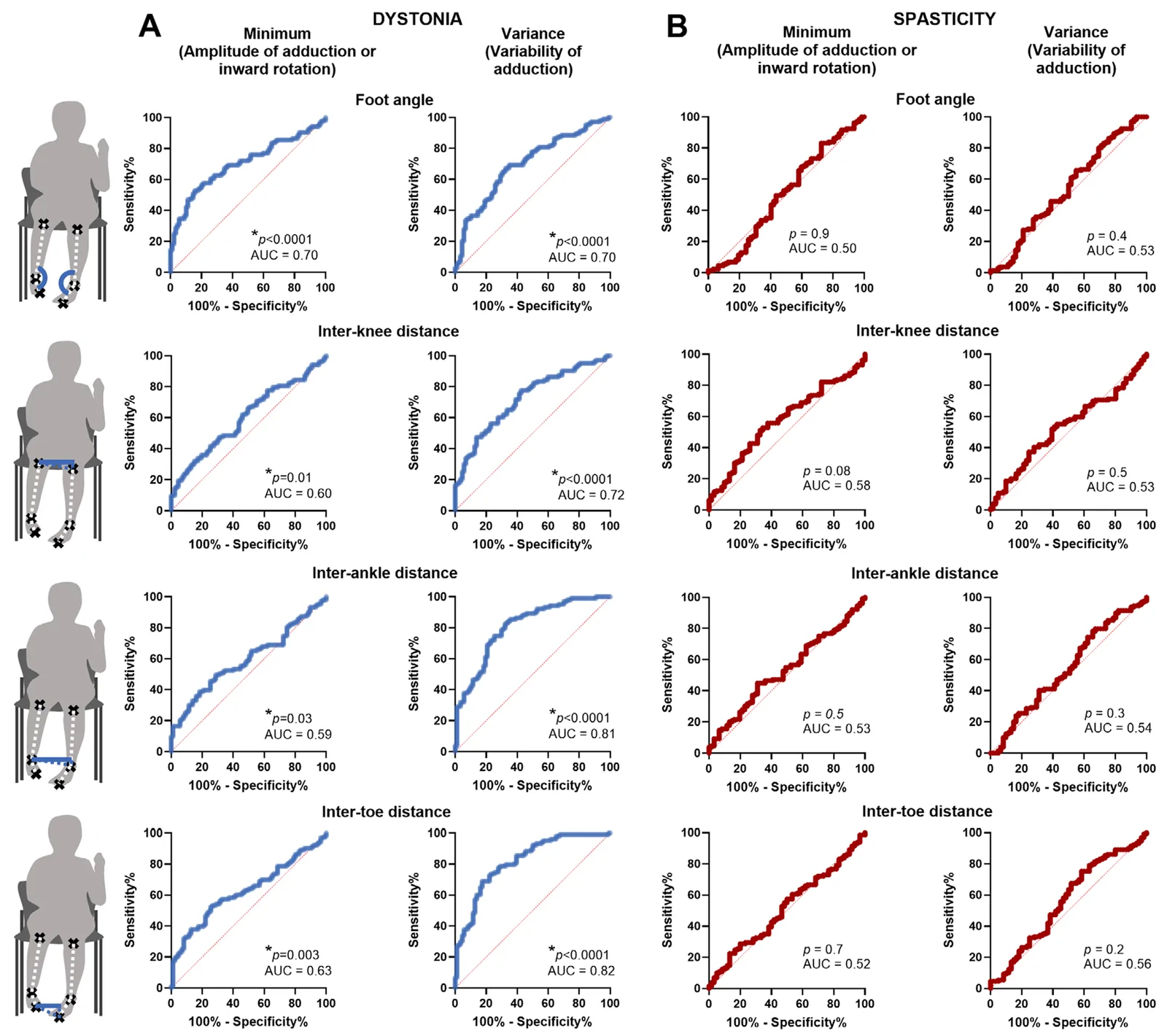
Discriminative ability of leg adduction variability and amplitude for leg dystonia. Metrics of leg adduction amplitude (left column) and variability (right column) are compared between people with cerebral palsy (CP) and leg dystonia (n = 103, average Global Dystonia Rating Scale [GDRS] of 1 or more) and without leg dystonia (n = 90, average GDRS of 0 to <1). Metrics are the minima (left column) and variances (right column) of the foot angle in the coronal plane (top row: angle between the knee, ankle, and toe labels), inter-knee distance (second row), inter-ankle distance (third row), and inter-toe distance (bottom row) with distance metrics calculated along an X-axis parallel to the floor and divided by the maximum knee-to-ankle distance to allow comparison across videos. (B) The same metrics of leg adduction amplitude and variability are compared between people with CP and leg adduction/inversion spasticity (n = 131, Modified Ashworth Score for hip adduction and foot inversion of 1 or more) and without leg adduction/inversion spasticity (n = 62, Modified Ashworth Score for hip adduction and foot inversion of 0). Receiver operator characteristic curves are shown with areas under the curves (AUC), *p < 0.05. [Color figure can be viewed at www.annalsofneurology.org]

**FIGURE 4:**
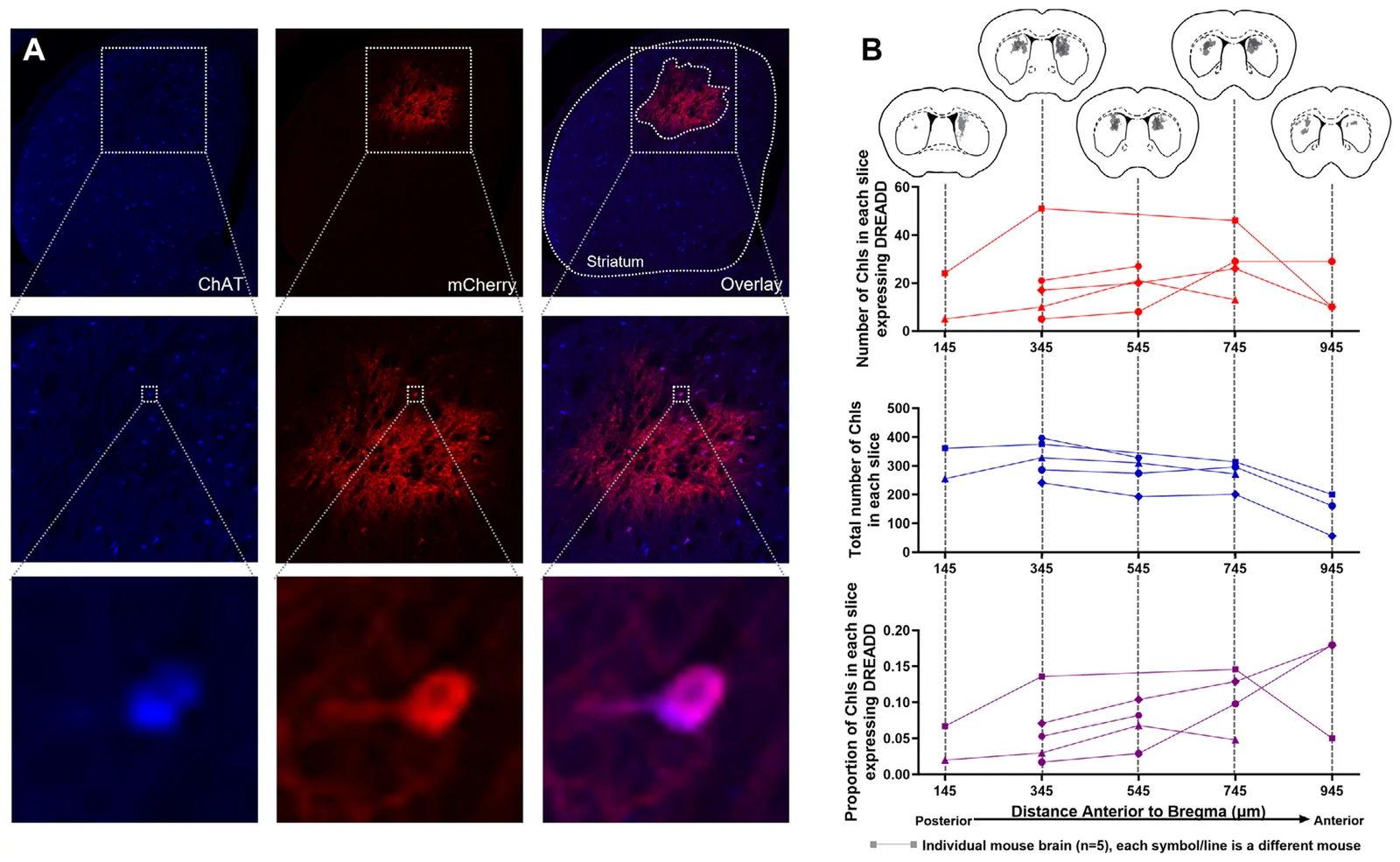
Localization and quantification of chemogenetically excited striatal cholinergic interneurons (ChIs). (A) Immunohistochemical staining for choline acetyl-transferase (ChAT) to identify ChIs (left column), mCherry to identify cells expressing the Gq designer receptors exclusively activated by designer drugs (DREADD) and associated mCherry fluorescent reporter (middle column), and overlay (right column). Rows successively show increased magnification of the striatal area of interest, showing that ChAT and mCherry co-express in the same neurons. (B) Mapping of the location and proportion of striatal ChI DREADD expression. Overlays of the regions expressing the DREADD are shown in representative brain slices (top), and parsed by number of striatal ChIs expressing the DREADD (top), total number of striatal ChIs in the slice (middle), and the ratio between these 2 values (bottom), between 145 and 945μm anterior to bregma. Data for 5 mice are shown, with each symbol/line representing a single mouse. [Color figure can be viewed at www.annalsofneurology.org]

**FIGURE 5:**
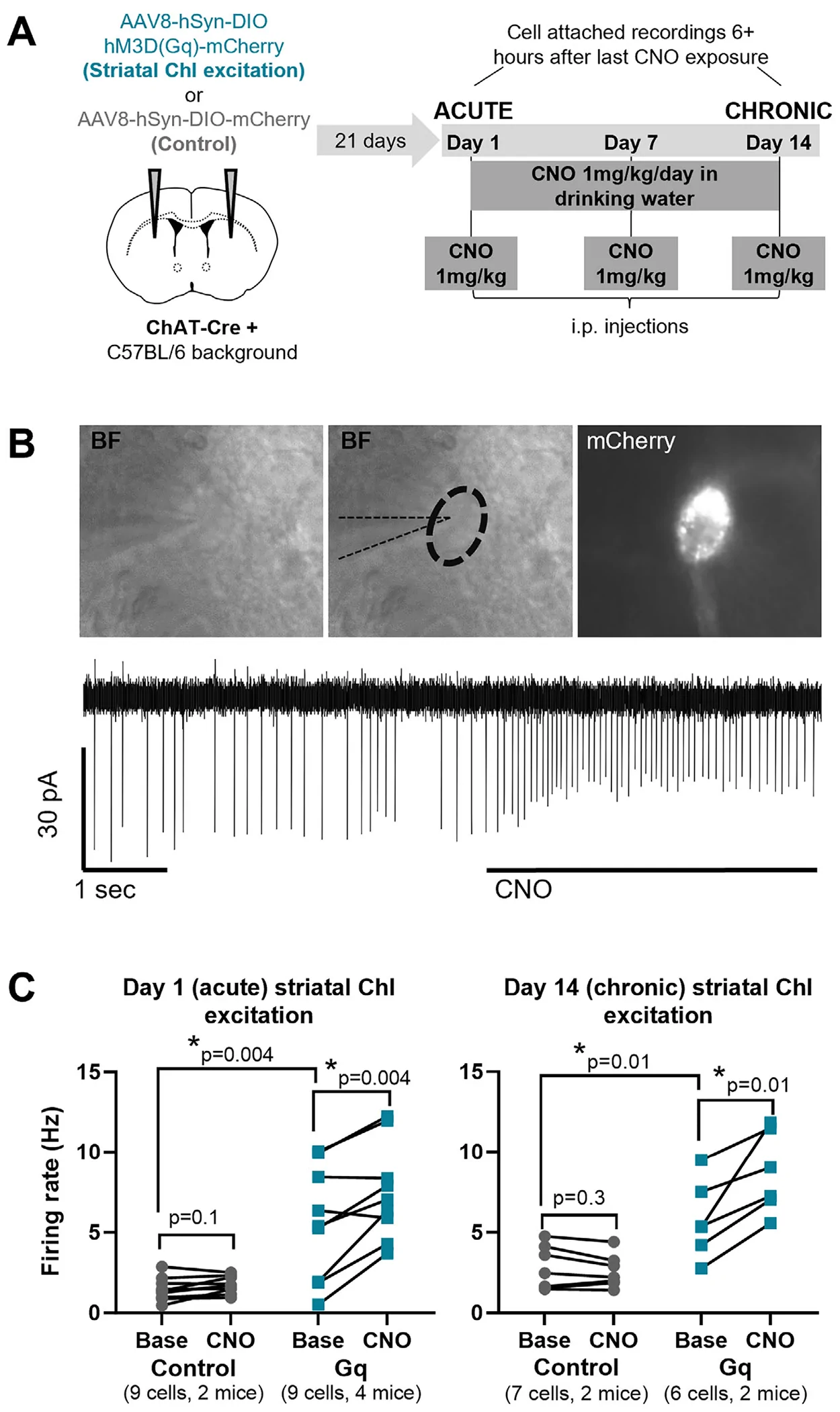
Demonstration of striatal cholinergic interneuron (ChI) excitation with clozapine-N-oxide (CNO). (A) Experimental paradigm showing generation of mice expressing the mCherry fluorescent tag in striatal ChIs (gray, control) and animals additionally expressing the Gq designer receptors exclusively activated by designer drugs (DREADD) (teal, experimental) and the shared 14 day CNO exposure protocol. (B) Example of cell-attached patching of a striatal ChI under brightfield (BF) and fluorescence to visualize the mCherry fluorescent tag. Cells that express the Gq DREADD increase their firing rate with bath application of CNO. (C) Striatal ChI firing rates and responses to bath-applied CNO determined with cell-attached recordings in mice on day 1 of the CNO exposure paradigm and day 14 of the CNO exposure paradigm, with baseline firing rates recorded at least 6 hours after the last CNO exposure. Paired *t* tests for comparing pre- and post-CNO firing rates; unpaired *t* tests for comparing baseline firing rates between groups, *p < 0.05. [Color figure can be viewed at www.annalsofneurology.org]

**FIGURE 6:**
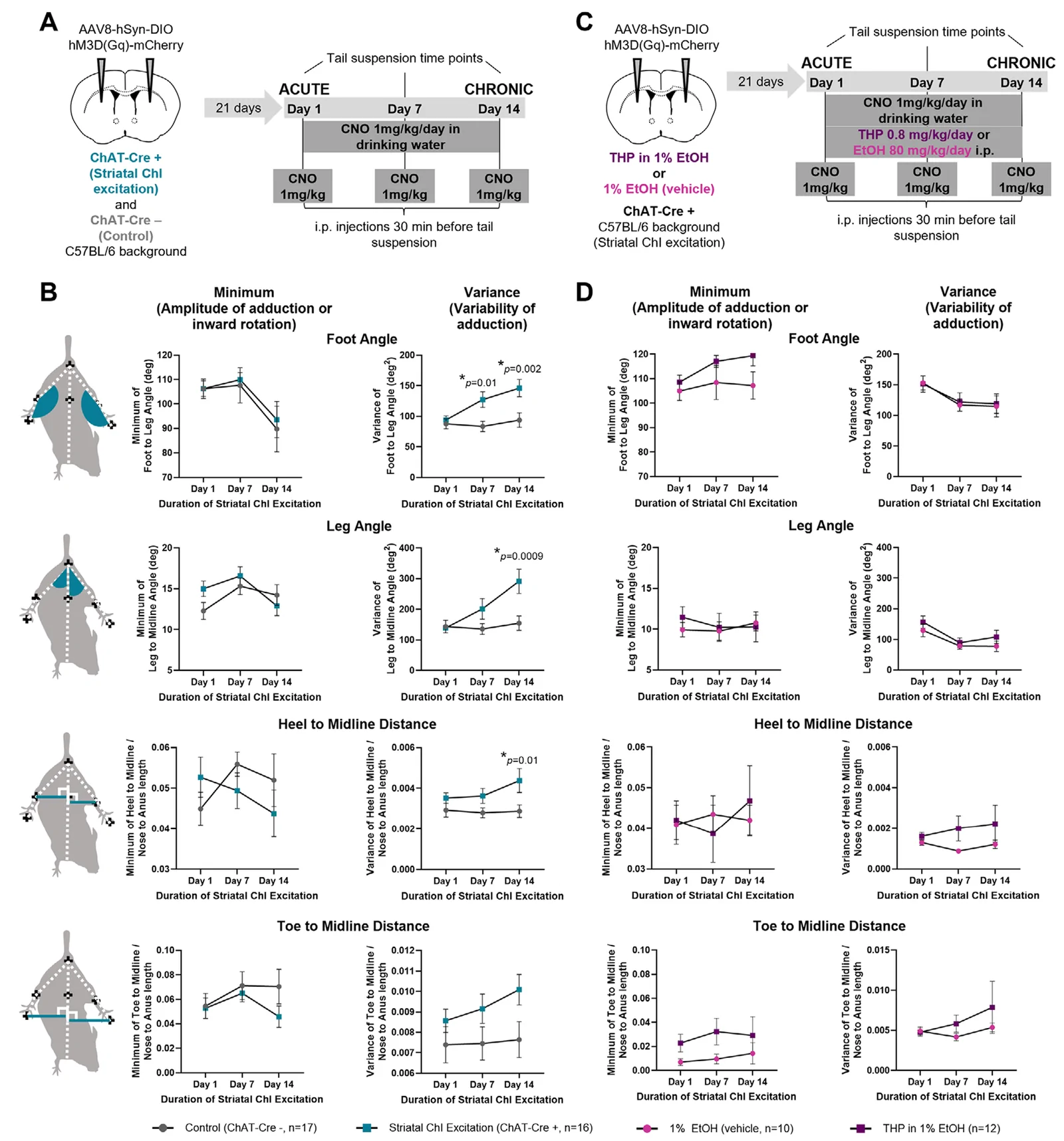
Leg adduction amplitude and variability in mice in response to 14 days of striatal cholinergic interneuron (ChI) excitation with and without ethanol and trihexyphenidyl treatment. (A) Experimental paradigm showing generation of mice expressing the Gq designer receptors exclusively activated by designer drugs (DREADD) (teal, choline acetyl-transferase (ChAT)-Cre positive) and their littermate controls who did not express the DREADD (gray, ChAT-Cre negative) and the shared 14 day clozapine-N-oxide (CNO) exposure and tail suspension protocol. (B) Experimental paradigm showing generation of mice expressing the Gq DREADD who received either 14 days of daily ethanol (EtOH, pink) or 14 days of trihexyphenidyl dissolved in ethanol (THP, purple) during a tail suspension protocol. (C) Metrics of leg adduction amplitude and variability are compared between mice who underwent chronic striatal ChI excitation (teal, n = 16) and mice who did not (gray, n = 17). Leg adduction metrics are the amplitudes or minima (left column) and variability or variances (right column) of the foot angle between the hindpaws and legs (top row), leg angle between the leg and the mouse midline (second row), distance between the heels and midline (third row), and distance between the toes and midline (bottom row). Distance metrics are calculated perpendicular to midline (drawn between the anus and trunk midpoint) and are divided by the maximum nose-to-anus distance to allow comparison across videos. Comparisons between mice that did and did not undergo ChI excitation are by repeated measures 2-way ANOVA with Bonferroni correction for multiple comparisons, *p < 0.05. (D) The same metrics are compared between mice who underwent chronic striatal ChI excitation who also received daily ethanol (pink, n = 10) or daily trihexyphenidyl dissolved in ethanol (purple, n = 12). Comparisons between mice that received ethanol and mice that received trihexyphenidyl are by repeated measures 2-way ANOVA with Bonferroni correction for multiple comparisons, *p < 0.05. [Color figure can be viewed at www.annalsofneurology.org]

**TABLE.** Demographic Characteristics of People with CP Assessed for Dystonia

	No dystonia (median GDRS <1), n = 89	Dystonia (median GDRS 1+), n = 104	Missing Data (n)
Age (yr, mean ± standard deviation)	7.6 ± 3.3	6.9 ± 2.9	6
Gestational age (weeks, mean ± standard deviation)	32.3 ± 6.4	31.6 ± 5.6	9
CP distribution (n, %)			
Hemiplegia	7, 8%	7, 7%	
Diplegia	66, 78%	53, 55%	
Triplegia	9, 11%	21, 22%	
Quadriplegia	3, 4%	15, 16%	
GMFCS (n, %)			
I	17, 22%	18, 19%	
II	36, 47%	36, 37%	
III	19, 25%	33, 34%	
IV	4, 5%	10, 10%	
V	0, 0%	0, 0%	
Leg spasticity (MAS summed across all leg ranges assessed) (n, %)			
No (MAS = 0)	7, 8%	8, 8%	
Yes (MAS ≥1)	80, 92%	91, 91%	
MAS 1–4	28, 32%	26, 26%	
MAS 5–9	24, 28%	32, 32%	
MAS 10–14	17, 20%	23, 23%	
MAS ≥15	11, 13%	11, 11%	
Leg spasticity (MAS summed for hip adduction and foot inversion only) (n, %)			
No (MAS = 0)	31,36%	31, 31%	
Yes (MAS ≥1)	56, 64%	69, 69%	
MAS 1–2	22, 25%	31, 31%	
MAS 3–4	23, 26%	24, 24%	
MAS 5–6	10, 11	11, 11%	
MAS ≥7	1, 1%	3, 3%	

Summed MAS scores for the legs include assessments of right and left hip flexion, hip adduction, knee extension, knee flexion, foot plantarflexion, and foot inversion with a possible score range of 0–48 if summed across all joint ranges or 0–16 if summed across just hip adduction and foot inversion).

CP = cerebral palsy; GDRS = Global Dystonia Rating Scale, with median score determined between 8 pediatric movement disorders specialists rating leg dystonia; GMFCS = Gross Motor Functional Classification System (I–II—independently ambulatory without assistive devices, III—independently ambulatory with assistive devices; IV–V—uses wheelchair for mobility); MAS = Modified Ashworth Scale (a 0–4 point scale indicating increasing severities of spasticity at each joint range of motion tested; yr = year.

## Data Availability

The authors confirm that the data supporting the findings of this study are available within the article, its supporting information, and in this online repository: https://wustl.box.com/s/07agml8jmw681et1bnsk2j4atghy5j9l.
