# Supplementary material for: Chronic Striatal Cholinergic Interneuron Excitation Causes Cerebral Palsy-Related Dystonic Behavior in Mice
Source: Ann Neurol. Author manuscript; Available in PMC 2026 Oct 29. (PMC13717702; doi:10.1002/ana.27299)
Supplement: Supplementary file 1 [file NIHMS2123102-supplement-Table_S1.docx]

**Supplementary Tables and Figures**

| **Feature selection method** | **Models discriminating between presence vs. absence of dystonia** | | **Models predicting dystonia severity** | |
| --- | --- | --- | --- | --- |
|  | Amplitude | Variability | Amplitude | Variability |
| Spearman's rho | 1 | 3 | 1 | 3 |
| Kendall's tau | 1 | 3 | 1 | 3 |
| Anova F-test /univariate feature selection | 1 | 3 | 1 | 3 |
| Maximum Relevance - Minimum Redundancy | 1 | 3 | 0 | 4 |
| ExtraTreesClassifier | 1 | 3 | - | - |
| Random forest | 1 | 3 | - | - |
| Sequential Feature Selection (forward) | 0 | 4 | - | - |

**Supplementary Table 1.** The top leg adduction metrics contributing to models discriminating between the presence versus absence of dystonia or to models predicting dystonia severity. Seven models (left) were used to identify the top 4 metrics out of 8 total (4 variability + 4 amplitude metrics) that could discriminate between the presence or absence of leg dystonia (middle) or predict leg dystonia severity (right). The number of variability or amplitude metrics that were selected as the top 4 metrics in each model is indicated.

**Supplementary Figure 1.** **Relationship between leg spasticity and leg adduction variability and amplitude. (A)** Metrics of leg adduction amplitude and variability are compared between people with CP and leg spasticity (red, n=131, Modified Ashworth Score for hip adduction, knee extension, knee flexion, foot plantarflexion, and foot inversion of 1 or more) and without spasticity (black, n=62, Modified Ashworth Score for hip adduction, knee extension, knee flexion, foot plantarflexion, and foot inversion of 0). Comparisons are by t-test, *p<0.05. Metrics are the minima (left column) and variances (right column) of the foot angle in the coronal plane (top row: angle between the knee, ankle, and toe labels), inter-knee distance (second row), inter-ankle distance (third row), and inter-toe distance (bottom row) with distance metrics calculated along an X-axis parallel to the floor and divided by the maximum knee-to-ankle distance to allow comparison across videos. **(B)** The same metrics of leg adduction amplitude and variability are compared between people with CP with and without leg spasticity using receiver operator characteristic curves. Areas under the curves (AUC) are shown, *p<0.05. **(C)** The same metrics of leg adduction amplitude and variability are correlated with Modified Ashworth Scores indicating leg spasticity severity in people with CP and leg spasticity (red, n=131, summed Modified Ashworth Scores for hip adduction, knee extension, knee flexion, foot plantarflexion, and foot inversion of 1 or more). Pearson correlation, *p<0.05.

**Supplemental Figure 2. Total movement of the heels (top row) and toes (bottom row) relative to the mouse midpoint. (A)** Total movement of mice that underwent striatal ChI excitation (teal, n=16) compared to mice that did not (grey, n=17). **(B)** Total movement of mice that underwent 14 days of striatal ChI excitation that also received daily ethanol (pink, n=10) are compared to mice that underwent 14 days of striatal ChI excitation that also received trihexyphenidyl dissolved in ethanol (purple, n=12). Comparisons are by repeated measures two-way ANOVA with Bonferroni correction for multiple comparisons, *p<0.05.
